# Hyperinsulinemia and Its Pivotal Role in Aging, Obesity, Type 2 Diabetes, Cardiovascular Disease and Cancer

**DOI:** 10.3390/ijms22157797

**Published:** 2021-07-21

**Authors:** Joseph A. M. J. L. Janssen

**Affiliations:** Department of internal Medicine, Division of Endocrinology, Erasmus Medical Center, 40, 3015 GD Rotterdam, The Netherlands; j.a.m.j.l.janssen@erasmusmc.nl

**Keywords:** hyperinsulinemia, insulin resistance, insulin secretion, insulin clearance, growth hormone, insulin-like growth factor-I, obesity, diabetes, cardiovascular disease, cancer, longevity

## Abstract

For many years, the dogma has been that insulin resistance precedes the development of hyperinsulinemia. However, recent data suggest a reverse order and place hyperinsulinemia mechanistically upstream of insulin resistance. Genetic background, consumption of the “modern” Western diet and over-nutrition may increase insulin secretion, decrease insulin pulses and/or reduce hepatic insulin clearance, thereby causing hyperinsulinemia. Hyperinsulinemia disturbs the balance of the insulin–GH–IGF axis and shifts the insulin : GH ratio towards insulin and away from GH. This insulin–GH shift promotes energy storage and lipid synthesis and hinders lipid breakdown, resulting in obesity due to higher fat accumulation and lower energy expenditure. Hyperinsulinemia is an important etiological factor in the development of metabolic syndrome, type 2 diabetes, cardiovascular disease, cancer and premature mortality. It has been further hypothesized that nutritionally driven insulin exposure controls the rate of mammalian aging. Interventions that normalize/reduce plasma insulin concentrations might play a key role in the prevention and treatment of age-related decline, obesity, type 2 diabetes, cardiovascular disease and cancer. Caloric restriction, increasing hepatic insulin clearance and maximizing insulin sensitivity are at present the three main strategies available for managing hyperinsulinemia. This may slow down age-related physiological decline and prevent age-related diseases. Drugs that reduce insulin (hyper) secretion, normalize pulsatile insulin secretion and/or increase hepatic insulin clearance may also have the potential to prevent or delay the progression of hyperinsulinemia-mediated diseases. Future research should focus on new strategies to minimize hyperinsulinemia at an early stage, aiming at successfully preventing and treating hyperinsulinemia-mediated diseases.

## 1. Introduction

Subjects with insulin resistance and hyperinsulinemia are at high risk of developing obesity, type 2 diabetes, cardiovascular disease, cancer and premature mortality [1,2,3,4,5,6,7,8,9]. The dogma has been for many years that insulin resistance is first and precedes hyperinsulinemia and type 2 diabetes mellitus. In this view, hyperinsulinemia was secondary and represented a compensatory mechanism to overcome systemic (peripheral) insulin resistance [10]. Insulin resistance was considered to be the primary etiological factor in the development of obesity, type 2 diabetes, cardiovascular disease and cancer, whereas the compensatory hyperinsulinemia was thought to be a direct consequence of insulin resistance [11]. However, recently the correctness of this view has been called into question [12,13,14]. It has even been proposed that hyperinsulinemia per se is primary and causes (and is not a consequence of) insulin resistance [13]. In this new concept, insulin resistance is proposed to be a physiological defense mechanism of the body that tries to prevent the development of hypoglycemia and to protect critical tissues from metabolic stress and nutrient-induced injury [15,16]. This opens the possibility that (early) interventions able to normalize/reduce plasma insulin concentrations might play a key role in the prevention and treatment of obesity, type 2 diabetes, cardiovascular disease, cancer and premature mortality [10,17]. This review focuses on the etiology of hyperinsulinemia and hyperinsulinemia-mediated disorders. It discusses how insulin might play a pivotal role in health, disease and longevity and (potential) strategies to prevent and manage hyperinsulinemia.

## 2. The Role of the Insulin–GH–IGF-I Axis in Healthy Subjects

**Figure 1:**
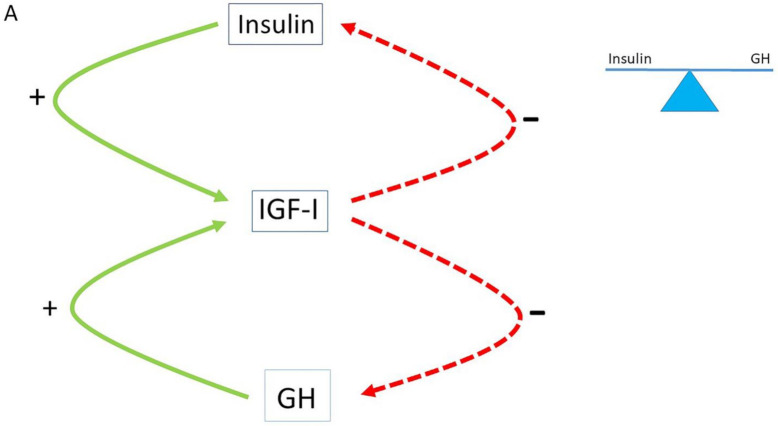

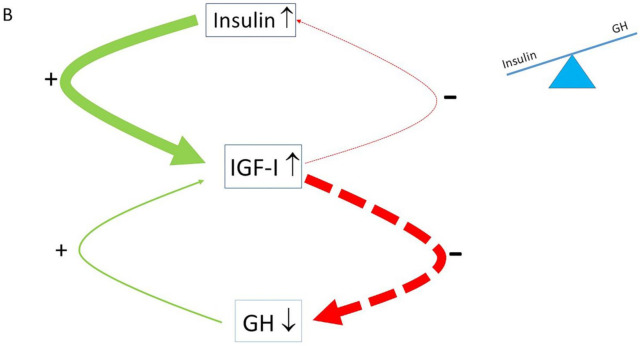
The insulin–growth hormone–IGF-I (insulin–GH–IGF-I) axis plays a central role in metabolism. (**A**) In healthy subjects, the insulin–GH–IGF-I axis is in balance: insulin and GH both stimulate IGF-I production in the liver, while after secretion IGF-I feeds back to suppress both insulin and GH. (**B**) The continuous food intake and energy surplus (Western diet) in modern societies has disturbed the normal balance of the insulin–GH–IGF-I axis. As a consequence, a shift of the insulin : GH ratio towards insulin (and IGF-I) and away from GH has occurred. The higher insulin : GH ratio lowers energy expenditure and induces fat accumulation. This insulin–GH shift promotes energy storage and lipid synthesis and hinders lipid breakdown, resulting in obesity due to higher fat accumulation and lower energy expenditure (see text for more details on how continuous foods intake and Western diet may influence the balance of the insulin–GH axis).

Insulin and insulin-like growth factor-I (IGF-I) show high homology [18]. They both belong to a phylogenetically ancient family that plays a fundamental role in the control of essential cellular and physiological processes such as the cell cycle, survival or apoptosis, cell migration, proliferation, differentiation, body growth, metabolism, reproduction and longevity [19]. The insulin and IGF-I molecules are both composed of an alpha and beta chain connected by disulfide bonds. Although insulin is in many respects structurally similar to IGF-I, insulin and IGF-I bind to distinct receptors. Insulin binds with high affinity to the insulin receptor whereas it has very low affinity to the IGF-I receptor, while the converse is true for IGF-I [18]. Despite these differences, both insulin and IGF-I secretion are coordinately regulated by changes in nutrient intake [20]. In addition, insulin delivery into the portal system is required for a normal hepatic IGF-I synthesis and bioavailability [21]. Insulin (produced in the pancreas) and GH (produced in the pituitary gland) both stimulate IGF-I production in the liver, while after secretion IGF-I feeds back to suppress both insulin and GH secretion [22,23]. In healthy subjects, the insulin–GH–IGF-I axis is in balance (Figure 1A).

## 3. The Role of the Insulin–GH–IGF-I Axis in Metabolism

There is much overlap in the endocrine, metabolic and anabolic effects of insulin, GH, and IGF-I, but all three hormones have divergent effects on glucose metabolism and insulin sensitivity (see below) [24]. Insulin controls glucose metabolism and glucose transport in the liver, muscles and fat cells [21]. Although glucose metabolism in the brain is largely non-insulin dependent, the infusion of insulin in the third cerebral ventricle suppresses endogenous hepatic glucose production independent of circulating insulin levels and other glucoregulatory hormones [25]. In the central nervous system, insulin may inhibit food intake and is required for regulating peripheral fat metabolism [21,26]. Pancreatic β-cells are often referred to as “fuel sensors” as they continually monitor and respond to dietary nutrients [27]. It is convenient to consider individual effects of different macronutrients on metabolism and insulin secretion. However, all macronutrients (carbohydrates, proteins and fat) work in concert and can stimulate insulin secretion. The intake of glucose (carbohydrates) is the primary stimulus for insulin secretion, but given the fact that β-cells are continually exposed to a complex milieu of many nutrients, it is the mixed nutrient sensing that generates the metabolic coupling factors (MCFs) involved in signaling for insulin secretion [27]. The so called “food and famine theory” proposed that during the day the metabolism in the human body shows a dynamic three-cycle phase [28] (Table 1). In the immediate postprandial period, insulin secretion induced by the intake of food is initially the major player in the three-cycle phase [29]. In this phase, insulin enhances glucose and amino acid uptake and stimulates the storage of these molecules by cells as well as lipid synthesis and storage [29]. In the early postprandial period, GH, insulin and IGF-I all stimulate anabolism (Table 1). Linked to the nutritional status of an individual, GH is the main stimulator of IGF-I secretion in the early postprandial period [9], whereas IGF-I increases insulin receptor sensitivity and suppresses proteolysis [29]. Moreover, IGF-I together with GH decreases protein oxidation [29]. In the late postprandial period, IGF-I attenuates GH-mediated insulin resistance, while it reduces insulin secretion by the pancreas [29] (Table 1). This promotes lipolysis and increases the combustion of lipids [29]. Thus, IGF-I in the late postprandial period plays a decisive role in the switch from glucose to free fatty acids (FFAs) as the main fuel in the body [29] (Table 1).

## 4. The Normal Balance between Insulin, IGF-I and GH Is Disturbed in Modern Societies

The normal balance between insulin, IGF-I and GH is associated with substrate and energy metabolism. In the prevailing evolutionary view, insulin promotes energy storage in the condition of energy surplus, whereas GH promotes lipid mobilization and oxidation when food is sparse [30]. However, in modern developed societies, continuous food intake, energy surplus (over-nutrition) and the consumption of high-sugar drinks often occur. As a consequence, the mean daily energy intake per person has increased (in the United States, for example, daily energy intake has increased since 1970 with 20% (+425 kcal/day) above the 2100 kcal/day) [31]. In addition, the “modern” Western diet is characterized by a high content of sugars, corn-derived fructose syrup, saturated fats and proteins but a reduced consumption of fruits and vegetables. Due to its composition, this “modern” Western diet may induce higher insulin secretion. Hyperinsulinemia per se in turn induces elevated IGF-I secretion. The elevated IGF-I subsequently suppresses GH secretion to lower levels than normal [32,33]. Moreover, insulin may also play an important role in regulating GH levels: insulin, like IGF-I, can suppress GH synthesis and release, while insulin is negatively correlated with GH levels in vivo [34,35]. Only a few days of overeating could markedly suppress GH secretion before any measurable weight gain [36]. It was suggested that the accompanying chronic hyperinsulinemia was a likely mediator of this rapid reduction in GH secretion [36]. In addition, hepatic GH resistance may develop due to chronic hyperinsulinemia [33,37]. As insulin has strong antilipolytic effects, hyperinsulinemia will initially result in excess fat accumulation. However, in the second instance, chronic hyperinsulinemia induces insulin resistance of the fat cell by downregulating the insulin receptor and/or diminishing other key downstream elements of the signaling pathway (see below paragraph “Loss of pulsatile insulin secretion contributes to insulin resistance” for further details). Insulin resistance of the fat cell will intensify lipolysis of the fat cell. This will induce a rise in the blood level of the FFAs, which in turn further decreases GH secretion [38]. Hyperinsulinemia in combination with decreased GH secretion causes a shift of the insulin : GH ratio towards insulin (as a consequence of increased insulin secretion and/or reduced hepatic insulin clearance) and away from GH (as a consequence of the suppression of GH secretion) (Figure 1B) [30]. This insulin–GH shift hinders lipid breakdown and promotes further energy storage and lipid synthesis and thereby promotes obesity [30]. Thus, an increased insulin : GH ratio is positively related to fat mass and negatively related to energy expenditure [30]. Acknowledging this pivotal role of insulin, IGF-I and GH in substrate and energy metabolism could be very important and essential for promoting health and preventing disease.

## 5. Methods to Measure Insulin

In clinical practice, the measurement of circulating insulin concentrations is not a routine part of diagnostic evaluation. Methods used to measure insulin and its precursors are bioassays, immunoassays, high-performance liquid chromatography (HPLC) and liquid chromatography–mass spectrometry (LC–MS/MS) [39,40]. The classical radioimmunoassay (developed by Berson and Yalow) measured not only insulin, but also proinsulin, the proinsulin conversion intermediates and any insulin derivatives produced by degradation, dimerization or glycosylation [41,42]. More recently, more specific immunometric assays and LC–MS/MS have been developed to measure more specific and sensitive insulin [41,43]. Although there are many commercially available insulin assays and despite the enormous number of improvements over the last 50 years in methodology, measuring insulin in blood is still associated with a significant number of analytical and clinical problems [41,43]. A reliable insulin measurement is essential for both clinical and research purposes. However, there is currently no good reference method for insulin assays available to compare insulin measurements from different manufacturers and clinical laboratories [39,43,44,45,46]. In addition, other important analytical pitfalls of insulin measurements in blood are related to hemolysis (may result in falsely low insulin values) and circulating anti-insulin antibodies (resulting in low insulin levels in some assays and high insulin levels in others) [45]. Insulin displays pulsatile secretion and insulin concentrations in blood oscillate with a periodicity of 5–15 min per oscillation, leading to significant changes in levels of plasma insulin in a short period of time [47,48]. Therefore, it is recommended to calculate the mean of three blood samples taken at 5 min intervals when a reliable fasting insulin level is required [48,49]. However, this is rarely performed in clinical practice and epidemiological studies [48]. After each meal, insulin secretion shows a short-lived peak [30]. It is important to realize that peripheral plasma insulin concentrations are affected by the kinetics of insulin distribution and degradation [50]. Following secretion by the pancreas, insulin is distributed into both the intra- and extravascular space. The volume of distribution of insulin is several times larger than the plasma volume [50]. A large fraction (up to 80%) of insulin secreted by the pancreas into the portal vein is cleared by the liver at first passage [51]. The half-life of insulin in the portal vein is estimated to be only ≈3–5 min [50,51,52]. Receptor-mediated insulin uptake followed by insulin degradation by hepatocytes is the basic mechanism of hepatic insulin clearance [53]. A decreased hepatic insulin clearance will result in peripheral hyperinsulinemia [47,54,55] (see below). Insulin and C-peptide are co-secreted in equimolar concentrations in a one-to-one molar ratio [54]. C-peptide is not extracted by the liver to any significant degree at first passage and is primarily cleared by the kidney. This is the reason that peripheral C-peptide concentrations reflect portal insulin secretion more accurately than the peripheral plasma insulin concentrations. Therefore, peripheral C-peptide concentrations are often used as a semi-quantitative marker of beta cell secretory activity in a variety of clinical conditions [56]. However, the longer half-life of C-peptide (≈35 min) versus insulin (3–8 min) favors the use of insulin to examine pulsatility, since the longer half-life of C-peptide will dampen oscillations and decrease signal-to-noise ratio oscillations correspondingly [47,57,58].

## 6. How to Define Circulating Hyperinsulinemia

Diagnosing hyperinsulinemia is not easy in clinical practice. There is no precise and universally accepted definition of hyperinsulinemia [59]. When cut-offs are available for hyperinsulinemia, these are in most cases based on fasting insulin levels [48]. The “normal” range of fasting insulin in healthy subjects varies considerably between labs, but has been reported to vary in a range between 3 and 30 µU/mL (18–180 pmol/L) [48]. In the National Health and Nutrition Examination Surveys (NHANES), fasting circulating insulin levels in healthy adult persons have been reported to be in a range between approx. 25 and 70 pmol/L [60]. Many studies define hyperinsulinemia based on arbitrarily chosen cut-off fasting insulin concentrations or 2 h insulin concentrations after an oral glucose load (for example, >67th percentile, >75th percentile or >90th percentile for non-diabetic subjects) [61,62]. In addition, as discussed above, laboratory standardization of insulin measurements remains a problem. It has been found that serum insulin measurement with different assays shows maximal 1.8-fold variation and therefore caution should be exercised when comparing results of insulin levels from different research labs/studies [46]. Moreover, differences in the circumstances of blood sampling and handling of blood samples before the actual measurement of insulin may further play a role in the variation of insulin measurements [3].

## 7. Loss of Pulsatile Insulin Secretion Contributes to Insulin Resistance

In healthy subjects, insulin secretion into the circulation is pulsatile and normally accounts for 75% of the daily insulin secretion [58]. It has been proposed that pulsatile insulin release may avoid the downregulation of (hepatocyte) insulin receptors compared to the constant delivery of insulin [63]. Prolonged near-physiological pulsatile insulin infusion has a greater hypoglycemic effect than continuous insulin infusion [64]. Moderate pulsatile hyperinsulinemia in nondiabetic human subjects does not induce insulin insensitivity [64]. This is in contrast to what is found after continuous hyperinsulinemia [64]. It has been demonstrated that hyperinsulinemia produced by continuous infusion of insulin (which increased plasma insulin concentrations to levels similar to those observed in insulin-resistant conditions) can produce insulin resistance (decreased insulin receptor sensitivity), and this decrease in insulin action may occur at the receptor and post-receptor level of the insulin receptor and is tissue-specific [65]. The loss of pulsatile insulin secretion is an early feature in the development of type 2 diabetes and may be involved in the (patho)genesis of insulin resistance in a variety of circumstances [65,66]. Intravenous delivery of insulin in a constant versus pulsatile pattern led to delayed activation of hepatic insulin receptor substrate (IRS)-1 and IRS-2 signaling, impaired activation of downstream insulin signaling effector molecules AKT and Foxo1, and decreased expression of glucokinase, suggesting that the physiological pulsatile pattern of insulin delivery is important for normal hepatic insulin signaling and glycemic control and essential to preserve insulin sensitivity [67]. In addition, several lines of evidence suggest that the pattern of insulin secretion by the pancreas determines hepatic insulin clearance: the liver preferentially extracts insulin delivered in pulses [68]. Therefore, the pulse mass of insulin release dictates both hepatic (directly) as well as extrahepatic (peripheral) insulin delivery [68]. Animal studies have also demonstrated the importance of pulsatile insulin delivery in the development of insulin resistance [16]. Although an acute rise in insulin is stimulatory for the insulin receptor, persistently and continuously elevated insulin levels desensitize the insulin receptor through multiple mechanisms, both at receptor and post-receptor level [12,69,70,71]. Continuous and long-term exposure to insulin causes a reduction in the number of insulin receptors at the cellular surface by promoting internalization as well as degradation of insulin receptors [69]. With continuous and long-term exposure to insulin, the kinase activity of the insulin receptor diminishes, probably because of combined effects of phosphorylation on serine residues on the insulin receptor, the dephosphorylation of tyrosines by the action of phosphatases, and the binding of inhibitory molecules [12,72,73,74,75]. All of these effects downregulate insulin receptor signaling and thereby cause insulin resistance.

## 8. Hyperinsulinemia Precedes Insulin Resistance

**Figure 2:**
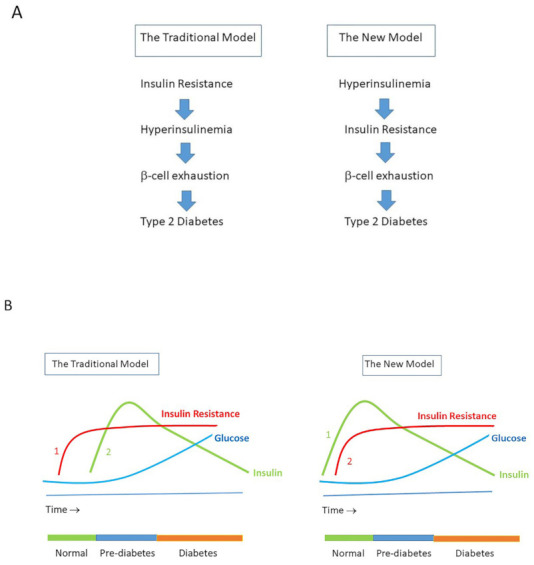
(**A**). The Traditional Model vs. The New Model of the pathogenesis of type 2 diabetes. The Traditional Model posits that insulin resistance is the primary abnormality leading to hyperinsulinemia, which is followed by β-cell dysfunction. When β-cells are no longer able to sustain sufficient insulin secretion to compensate hyperglycemia, they become exhausted and frank type 2 diabetes will develop. The Traditional Model has been called into question since it cannot explain that hyperinsulinemia can already be found in subjects with normal glucose tolerance (see text for details). In the (alternative) New Model, chronic hypersecretion of insulin (due to genetics and environmental factors) is the primary abnormality leading to hyperinsulinemia. Hyperinsulinemia initiates and sustains the development of insulin resistance (and obesity), until the β-cells fail and become exhausted and ultimately frank type 2 diabetes develops. (**B**) The Traditional Model vs. The New Model of the pathogenesis of type 2 diabetes. In the Traditional Model, insulin resistance (1) precedes hyperinsulinemia (2), which is followed by β-cell exhaustion and finally frank type 2 diabetes. In the New Model, hypersecretion of insulin and the resulting hyperinsulinemia (1) primarily cause insulin resistance (2), which is followed by β-cell exhaustion and finally frank type 2 diabetes. Note that in The New Model hyperinsulinemia is already present when there is a normal glucose tolerance.

In healthy subjects, plasma glucose is maintained within narrow ranges by a classic negative feedback system [76]. After meals, insulin secretion by the pancreas is stimulated by a rise in glucose and this brings plasma glucose back to baseline [76]. In this negative feedback system, insulin secretion is controlled and glucose levels remain within the normal range as long as subjects are able to overcome insulin resistance by increasing insulin secretion [76]. However, as soon as subjects have lost this ability, they will progress to impaired glucose tolerance and/or type 2 diabetes [76]. Until very recently, the prevailing view was that insulin resistance (that is, resistance to insulin’s role in promoting glucose uptake by muscle and fat cells) preceded and caused hyperinsulinemia [77]. In this view, insulin resistance was the initial defect leading to the development of metabolic syndrome, hyperglycemia and type 2 diabetes after years or even decades later [78]. However, in genome-wide association studies (GWAS), only a few loci point to insulin resistance as the primary cause of type 2 diabetes, while the majority of the loci identified by GWAS point towards defects of the β-cell of the pancreas [55,79]. This raises the distinct possibility that a β-cell defect in insulin secretion that initially causes inappropriate hypersecretion of insulin at basal plasma glucose concentrations may be a driver of insulin resistance by insulin-induced downregulation of insulin receptors [13,16]. It is important to realize that the actual diagnostic criteria for normal glucose tolerance, impaired glucose tolerance and type 2 diabetes are not defined on the basis of pathophysiological abnormalities [80]. Thus, although cross-sectional studies have postulated that insulin secretion follows an inverted U-pattern (also termed Starling’s curve of the pancreas) during natural progression from normal glucose tolerance to impaired glucose tolerance and type 2 diabetes [72], it has been repeatedly reported that a large part of individuals with normal glucose tolerance already show hyperinsulinemia before the development of impaired glucose tolerance/obesity. For example, Ferrannini et al. found increased plasma insulin levels in subjects with normal or near normal glucose tolerance [80]. In addition, it is difficult to understand how insulin resistance in subjects with normal glucose tolerance could be responsible for increased insulin secretion when blood glucose concentrations are still within the normal range. This is another argument against a primary role for insulin resistance-mediated hyperinsulinemia [81]. In addition, if fat cells were already resistant to insulin in the early phase, it is difficult to understand how hyperinsulinemia could stimulate lipogenesis and induce obesity by driving calories into fat cells [82]. Recently, a new model has been brought forward: in this new model, hyperinsulinemia is considered the primary event that secondarily causes insulin resistance and type 2 diabetes [12,81] (Figure 2). In support of this new model, insulin secretion has been found to be elevated before the development of hyperglycemia in a longitudinal study of Rhesus monkeys, developing a form of type 2 diabetes, which appears to be very similar to that found in humans [83]. In addition, an increasing number of human studies support the hypothesis that basal hyperinsulinemia is primary and that it contributes secondarily to insulin resistance and many diseases and conditions [12] (Table 2). 

**Figure 3:**
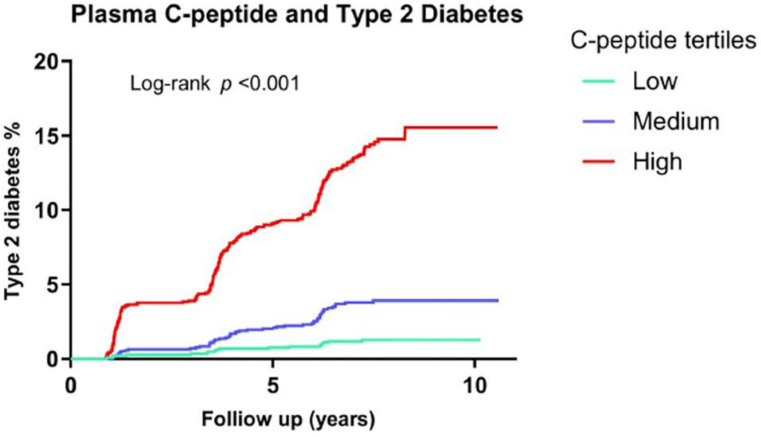
Kaplan–Meier curves for the development of diabetes according to sex-specific tertiles of C-peptide at baseline in 5176 participants of the PREVEND study. Low = M: <642, F: <592 pmol/L; medium = M: 642–890, F: 592–803 pmol/L; high = M: >890, F: >803 pmol/L; PREVEND: Prevention of Renal and Vascular End-Stage Disease. Note that in this population-based cohort, C-peptide levels in the highest tertile were associated with a higher incidence of type 2 diabetes during a median follow-up of 7, 2 years, compared to lower C-peptide levels (*p* < 0.001), and this association was independent of age, sex, BMI, family history of diabetes, blood pressure, triglycerides, HDL cholesterol, and fasting plasma glucose. Reproduced from S. Sokooti, L.M. Kieneker, M.H. de Borst, A. Muller Kobold, J.E. Kootstra-Ros, J. Gloerich, A.J. van Gool, H.J. Lambers Heerspink, R.T. Gansevoort, R.P.F. Dullaart, S.J.L. Bakker. Plasma C-Peptide and Risk of Developing Type 2 Diabetes in the General Population. J Clin Med. 2020 Sep 17; 9(9):3001. doi:10.3390/jcm9093001.

In a prospective population-based study among subjects with normal glucose tolerance at baseline, subjects with the highest C-peptide at baseline showed the highest incidence of type 2 diabetes during a follow-up of 7.2 years (Figure 3, next page) [84]. 

In another study, half of the 4485 subjects showed hyperinsulinemia despite normal glucose clearance, suggesting that hyperinsulinemia likely occurs as a “silent disease” in a substantial proportion of an otherwise healthy population [85]. In Pima Indians with normal glucose tolerance, evidence was found that fasting hyperinsulinemia itself, independent of a low rate of insulin-stimulated glucose uptake, predicted the cumulative incidence of type 2 diabetes during a 7 year follow-up [86]. Therefore, the authors concluded that high fasting plasma insulin concentrations were not a reflection of insulin resistance but rather the consequence of a basal hypersecretion of insulin relative to the degree of insulin resistance [86]. In a fourth study, the prevalence of insulin hypersecretion in nondiabetic, normotensive obese women exceeded the prevalence of insulin resistance, which was relatively low [87]. 

In a fifth study, a greater insulin-induced secretory response was found in subjects with normal glucose tolerance after pre-exposure to hyperinsulinemia, and this latter effect was independent of insulin sensitivity: stimulatory effects on insulin secretion were even stronger in insulin-sensitive individuals than in insulin-resistant individuals [88]. Moreover, it has been found that hyperinsulinemia drives adipose tissue inflammation in obese mice, suggesting that insulin may also impair systemic insulin sensitivity by specifically enhancing adipose inflammation [89]. Interestingly, the concept has been put forward that the insulin resistance of tissues can be considered as a physiological “defense mechanism” of cells against metabolic stress in response to chronic over-nutrition/adiposity [90]. In this concept, it was postulated that tissues normally responsive to insulin for glucose uptake (such as muscle and fat cells) are protected from chronic hyperinsulinemia-mediated nutrient excess (intracellular hyperglycemia) due to the development of (hyperinsulinemia-induced) insulin resistance [35].

## 9. Which Factors Cause Hyperinsulinemia?

**Figure 4:**
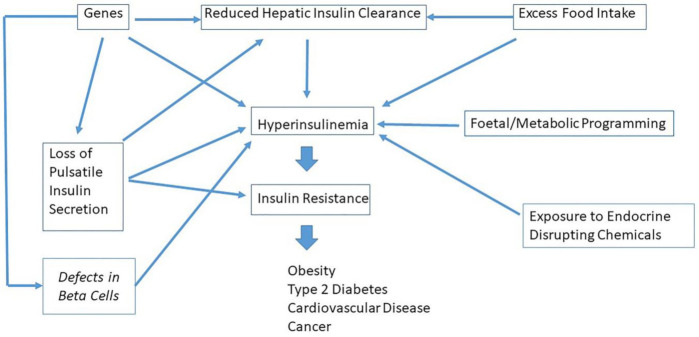
Some factors involved in hyperinsulinemia. Certain genes, hepatic insulin clearance, excess food intake, quality of nutrition, fetal/metabolic programming and exposure to endocrine disrupting chemicals have all been suggested as possible etiological factors in the development of hyperinsulinemia. Loss of pulsatile insulin secretion may contribute to changes in hepatic insulin clearance, hyperinsulinemia and insulin resistance. Note: genes, excess food intake and loss of pulsatile insulin secretion may modify hepatic insulin clearance (see also text for more details).

Genetic, environmental, and dietary factors have been associated with hyperinsulinemia [91]. As discussed above, there is mounting evidence that hyper-responsiveness of the β-cell may be primary [90]. It was demonstrated in Pima Indians that hyperinsulinemia is a highly heritable trait aggregating in families [92]. This strongly suggests that genetic factors determine whether a person becomes hyperinsulinemic [86]. Further support for a contribution of genetic factors in the development of hyperinsulinemia was found in a study of healthy twin pairs, which showed heritability for fasting insulin concentrations and 30-min insulin concentrations after an oral glucose load (54% in women and 37% in men; 57% in women and 47% in men, respectively) [93]. In 2011, during her Banting Lecture Barbara Corkey proposed a model in which environmental factors induce elevated levels of insulin superimposed on a susceptible genetic background of basal (hyper)insulinemia [13]. In this model, excess nutrient ingestion stimulates insulin secretion, fat storage and consequent insulin resistance [13]. Infusions of FFAs in normal human subjects induced an increase in circulating insulin levels [94]. It has been further shown in vitro that 18-h exposure to FFAs of isolated β-cells of the pancreas induces elevated insulin secretion [95]. Monoglycerides are commonly added in small quantities to commercial food products. It has been demonstrated in vitro that mono-oleoylglycerol stimulates insulin secretion at basal glucose concentrations of 3.0 mmol/L [95]. Moreover, artificial sweeteners (such as saccharin) also stimulated insulin release in isolated pancreatic β-cells [96]. Reactive oxygen species (ROS) are chemically reactive molecules containing oxygen [13]. Although ROS in high amounts can cause damage known as oxidative stress to cells and disturb insulin signaling, it also forms as a natural byproduct of metabolism. Previous studies showed that a modest ROS production functions as a signal in glucose-stimulated insulin secretion [13,97]. Several compounds that stimulate basal insulin secretion also generate ROS: both mono-oleoylglycerol and saccharin compounds also stimulate insulin secretion effectively by generating a small increase in ROS [13]. Evidence further points to a stimulating role of amino acids in insulin secretion [98]. Arginine and branched-chain amino acids such as leucine can function as powerful insulin secretagogues [99,100]. Environmental factors during intrauterine development may permanently alter the structure, physiology and metabolism of the body [101]. A substantial body of evidence suggests that an abnormal intrauterine milieu elicited by maternal metabolic disturbances as diverse as undernutrition, placental insufficiency, diabetes or obesity of the mother may program susceptibility in the fetus to develop hyperinsulinemia and obesity, diabetes, hypertension and cardiovascular diseases later in life [102]. In addition, it has been found in animal models that exposure to a high-carbohydrate milk formula during the suckling period results in permanent metabolic programming of hyperinsulinemia throughout adulthood [103]. Although more experimental work and prospective longitudinal studies are needed, there is accumulating evidence suggesting that exposure to endocrine disrupting chemicals may play a pathophysiological role in the development of hyperinsulinemia [104]. In rodents, for example, bisphenol, an endocrine-disrupting chemical, increases pancreatic insulin content and favors postprandial hyperinsulinemia [105]. Collectively, from the data presented above, it can be concluded that both basal and stimulated insulin secretion are under genetic and multifactorial control and can be modulated through numerous factors [106]. Which other mechanisms could lead to hyperinsulinemia? Insulin levels are determined by a balance between insulin release and disappearance. As discussed above, 20–80% of the secreted insulin is cleared by the liver and never enters the systemic circulation. It has been further found that there is a wide variation in hepatic as well extrahepatic clearance in human subjects without diabetes [107]. It is well documented that Afro Americans (AAs) show an increased risk for type 2 diabetes compared with European Americans (EAs) [55]. Hepatic clearance of insulin in children 7–13 years of age was already significantly lower in AAs vs. EAs [55]. Adult AAs tend to have higher plasma insulin concentrations than EAs and it is very plausible that this may be related to differences in hepatic insulin clearance: after an overnight fast, hepatic insulin first-pass extraction in AAs was one third compared to EAs [108]. Although the mechanism underlying the reduced insulin degradation by the liver was not clear, Bergman et al. hypothesized that peripheral hyperinsulinemia in the prediabetic situation results from reduced hepatic insulin clearance rather than the overproduction of insulin by pancreatic islets [55]. In this concept, the stimulating effects of hyperinsulinemia in the periphery are dampened by the development of insulin resistance in muscles and fat cells, the latter directly caused by an overexposure of the peripheral tissues to endogenous insulin [55]. The peripheral insulin resistance causes extra stress for the β-cells of the pancreas, and over time this may result in their ultimate failure and development of frank type 2 diabetes [55,108]. Experimental evidence showed that hepatic insulin clearance indeed can be a major and immediate regulator of systemic insulin concentrations [109]. Hepatic insulin clearance can decrease within days when a person switches to a diet with an increased energy intake that in particular contains carbohydrates [109]. On the other hand, it has been suggested that hepatic insulin clearance is a highly heritable trait and several chromosomal loci have been identified that harbor genes regulating insulin clearance [110]. Hepatic insulin-degrading enzyme (IDE) activity is a strong controller of systemic insulin levels [111,112]. Therefore, hepatic IDE impairments may also be a strong driver of hyperinsulinemia [113]. Recently, robust genetic evidence was provided that IDE controls insulin clearance in men and regulates postprandial glucose excursions [113]. Moreover, this genetic control was found abrogated in prediabetes [113]. It was further suggested that IDE polymorphisms governing postprandial hepatic insulin clearance are likely to be affected by epigenetic modifications induced by hypercaloric diets, leading to an impaired capacity to fine-tune postprandial insulin levels [113]. Epigenetic processes acquired early or later in life, β-cell size and mass, β-cell insulin receptor expression, differences in metabolic clearance of insulin, differences in the insulin constitutive secretory pathway, differences in central (hypothalamic) regulatory pathways and/or the activity of the parasympathetic nervous system have all been suggested as potential factors involved in the development of hyperinsulinemia [86]. Which specific genetic and exogenous factors determine hyperinsulinemia in vivo in humans is not clear at present (Figure 4).

## 10. Hyperinsulinemia Is a Common Etiological Factor in Many Diseases

**Figure 5:**
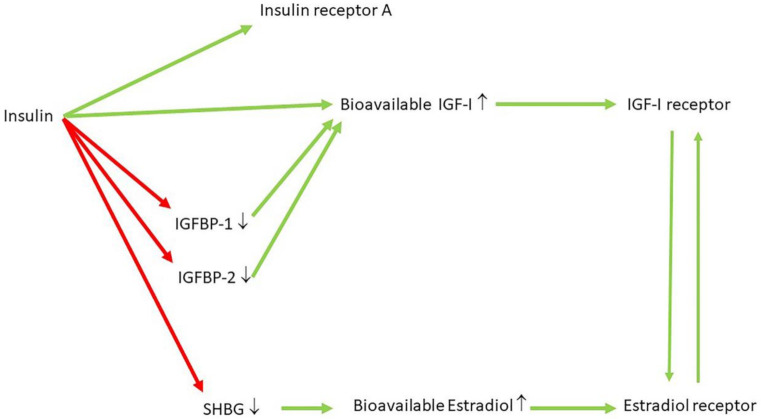
Interactions between insulin, IGFBP-1, IGFBP-2, SHBG, IGF-I bioavailability, the insulin receptor-A, the IGF-I receptor, estradiol and the estradiol receptor. Inhibitory effects are shown in red and stimulatory effects in green. Insulin stimulates the insulin receptor A (directly) and increases bioavailable IGF-I and estradiol (indirectly). Note that there is also bidirectional cross-talk between the IGF-I receptor and the estradiol receptor. IGFBP-1 = insulin-like growth factor binding protein-1, IGFBP-2 = insulin-like growth factor binding protein-2, SHBG= sex hormone binding globulin, ↑ levels increase, ↓ levels decrease.

There is strong evidence that hyperinsulinemia is an important precursor to obesity [91,114]. The hypersecretion of insulin at normal basal glucose concentrations may lower blood glucose, thereby stimulating eating [13]. This may increase fat mass [13]. In support of this, genetic predisposition to higher glucose-stimulated insulin secretion in adults was associated with higher BMI [115]. While initially insulin sensitivity was comparable between obese and normal children, early in the evolution of obesity, both insulin and C-peptide secretion to a normal meal were found increased in obese children compared to normal children [116]. Conversely, the suppression of diet-induced hyperinsulinemia in growing female mice provides long-term protection against obesity [117]. In addition, chronic hyperinsulinemia reduces insulin sensitivity and metabolic functions of brown adipocytes, and this leads in mice to increased body weight gain, fat mass and impaired glucose intolerance with reduced energy expenditure and insulin sensitivity [118]. In a population of 1168 adults and adolescents with normal glucose tolerance at baseline, primary insulin hypersecretion was associated with a worse clinical and metabolic phenotype and predicted deterioration of glucose control after 3 years follow-up, and this relationship was independent of insulin resistance [119]. Longitudinal studies performed in Micronesian Nauruans [120], American Pima Indians [121], Mexican-Americans [122], Nauruans [123] and Europoids [124] have demonstrated that hyperinsulinemia in the presence of normal glucose can predate the development of type 2 diabetes by many years. Furthermore, in subjects with metabolic syndrome, early phase insulin secretion was increased independently from insulin sensitivity, suggesting that hyperinsulinemia might be the primary defect contributing to glucose intolerance [125]. In the Israel Study of Glucose Intolerance, Obesity and Hypertension, basal hyperinsulinemia in normoglycemic adults conferred an increased risk for the development of impaired glucose regulation after more than two decades follow-up [126]. In another study, the 25% of the subjects with the highest insulin response to a glucose challenge at baseline had after 15 years follow-up an increased incidence of impaired glucose tolerance and type 2 diabetes [127]. Moreover, Mendelian randomization studies showed that individuals carrying ≥17 alleles that raise fasting insulin levels have an increased risk of elevated blood pressure, cardiovascular disease and type 2 diabetes [128]. Apparently healthy persons with hyperinsulinemia and a normal glucose tolerance showed an increase in risk factors for coronary artery disease, as compared with a well-matched group of healthy subjects with normal insulin levels [129]. Many prospective studies in nondiabetic subjects have found that elevated insulin levels, either fasting or in response to oral glucose, have a predictive role in the development of cardiovascular disease [2,130,131,132,133]. This association was independent of the effects of other well-known cardiovascular risk factors. Hyperinsulinemia may play a causal role in atherosclerosis [133]. Insulin may stimulate smooth muscle cell proliferation and migration and enhance lipoprotein metabolism [134,135,136,137,138]. In support of a role of hyperinsulinemia in atherosclerosis, it was recently reported that higher insulin levels in subjects with normal glucose tolerance were the best predictor of restenosis after previous revascularization by percutaneous coronary intervention because of ischemic heart disease [139]. Hyperinsulinemia could further contribute to hypertension by stimulating basal sympathetic tone and renal sodium reabsorption [140,141]. However, evidence that reducing hyperinsulinemia prevents atherosclerosis is lacking at this moment. There are clear, direct links between hyperinsulinemia, hypertriglyceridemia and non-alcoholic fatty liver disease [142]. Moreover, hyperinsulinemia may cause the activation of the hypothalamus–pituitary–adrenal axis [143]. Many epidemiological studies also support a role for hyperinsulinemia in cancer [144]. A meta-analysis demonstrated a clear link between high insulin levels and colorectal cancer (RR 1.35; 1.13–1.61) and pancreatic cancer (RR 1.70; 1.10–2.63) [145]. The Prospective Women’s Health Study measured serial insulin levels over a six-year period and showed a two-fold increased risk of breast cancer in subjects with insulin levels in the top tertile compared with the bottom [146]. In many cancer cells, the insulin receptor is overexpressed and the insulin receptor A isoform, which has predominant mitogenic effects, is more dominantly represented than the insulin receptor B isoform [147,148]. The increased expression of the insulin receptor A isoform in cancer cells probably provides a selective growth advantage to malignant cells exposed to hyperinsulinemia [147,148] (Figure 5). In addition, hyperinsulinemia also increases IGF-I bioavailability through the downregulation of insulin-like growth factor binding protein-1 (IGFBP-1) and IGFBP-2, which both may inhibit IGF-I actions [148,149] (Figure 5). Moreover, insulin inhibits the hepatic synthesis of sex-hormone binding globulin (SHBG) and stimulates the ovarian synthesis of sex steroids, which may promote cellular proliferation by increasing bioavailable estrogens in breast epithelium and endometrium [150,151] (Figure 5). Furthermore, patients with hyperinsulinemia, hyperglycemia and insulin resistance may have an increased risk of cancer due to the overproduction of reactive oxygen species (ROS) that can damage DNA and thereby contribute to mutagenesis and carcinogenesis [150]. For this reason, conditions with hyperinsulinemia (prediabetes, metabolic syndrome, obesity, type 2 diabetes before pancreas exhaustion) have been associated with increased cancer risk and progression [8,147,152].

## 11. The Role of Insulin (Signaling) in Longevity

**Figure 6:**
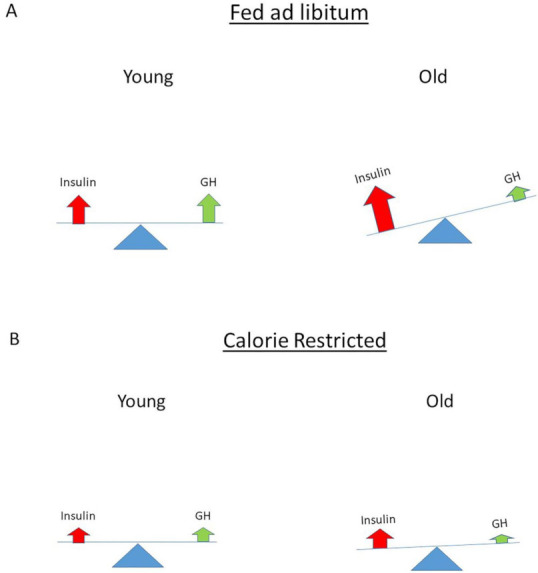
In the view of Parr, nutritionally driven “normal” insulin exposure is central to imbalance of the insulin–growth hormone axis (insulin–GH axis) [157,158]. (**A**) At young age there is balance in the insulin–GH axis. However, when there is (excessive) food intake ad libitum, a progressive imbalance develops in the insulin–GH axis with aging. This imbalance is the direct consequence of a food-induced increase in insulin secretion and the five-fold decline in GH secretion, which normally occurs between the age of 18 and 80. Due to the progressive imbalance in the insulin–GH axis with aging the decline in reserve capacity of cells and organ functions is accelerated. (**B**) Calorie restriction started at a young age will cause (a lifelong) lower insulin and GH secretion. As a consequence of calorie restriction, cumulative lifetime exposure to insulin at the same age will be less and lifespan will be extended by better maintaining the balance in the insulin–GH axis: this will result in long-term low (but functional) insulin levels and a high insulin receptor sensitivity (see text for more details).

There is evidence for an important role of insulin signaling in the control of aging and longevity. Disruption of the insulin signaling pathway extends lifespan in several animal models and collectively these data suggest the distinct possibility that hyperinsulinemia might play a pivotal role in the aging process [153]. In 1956, Harman proposed the free-radical theory of aging, which postulated that aging is caused by cumulative oxidative damage to cells by oxidative stress (i.e., free radicals produced during aerobic respiration) [154]. Hyperinsulinemia may be the missing link among oxidative stress, aging and age-related diseases. Independently from hyperglycemia, hyperinsulinemia could enhance oxidative stress by facilitating protein oxidation and by stimulating free radical generators and anti-oxidative enzymes [155]. In addition, it has been suggested that another pro-aging effect of insulin involves the inhibition of the proteasome since insulin has major effects on cellular protein turnover by inhibiting protein degradation [155]. The higher the insulin levels, the lower the proteasome activity and, presumably, the faster the accumulation of oxidized proteins in the body [155]. In support of this latter mechanism, mutations reducing overall insulin signaling in Caenorhabditis Elegans, Drosophila Melanogaster and other animal models decrease oxidative stress and the accumulation of oxidized proteins and extend lifespan [155,156]. Previously, Parr hypothesized that nutritionally driven insulin exposure controls the rate of mammalian aging [157]. In his concept, insulin occupies a central position for three reasons: (1) insulin regulates the level of bioavailable IGF-I, which controls overall growth and proliferation; (2) insulin levels (indirectly) modulate mitochondrial energy production and oxidative stress; and (3) insulin levels affect the insulin–GH hormonal axis balance [157]. In the view of Parr, there is a balanced insulin/GH secretion at young adulthood generating sufficient bioavailable IGF-I for tissue maintenance and growth events [158] (Figure 6A). 

However, after the third decade of life, there is a progressive decline of GH secretion with “normal” to high insulin [158]. With aging, this causes a progressive imbalance in the insulin–GH axis, leading to a gradual decline in cell and organ functions and consequent rise of diseases [158] (Figure 6A). Parr hypothesized that caloric restriction (CR) or halving insulin levels by increasing insulin sensitivity restores the balance of the insulin–GH axis and thereby slows down the loss of the physiological reserve capacity that permits a longer lifespan [158] (Figure 6B). CR has been demonstrated to extend normal lifespan and improve health in many animal studies [159]. CR retards age-related physiological decline and prevents the incidence of age-related diseases [160]. CR results in a higher insulin sensitivity and lower but functional insulin and IGF-I levels, and this may slow down age-related physiological decline and the incidence of age-related diseases [158]. Lower insulin and IGF-I levels will limit oxidative stress and cellular damage caused by the toxic byproducts of metabolism, while enough resources will be available for preserving and maintaining normal functions of the body [158]. Reduced exposure to insulin and IGF-I after CR may account for most, but not all, of the life extension benefits of CR, and this may be equally applicable to all mammals and humans [158]. Interestingly, in the Baltimore Aging Longitudinal Study of Aging, men with lower plasma insulin levels had a greater survival than subjects with higher insulin levels [161]. In addition, significantly lower C-peptide concentrations with a normal glucose tolerance have been reported in healthy Italian centenarians compared to normal adults and elderly subjects, and these centenarians demonstrated exceptional sensitivity to insulin and low circulating insulin levels, (again) suggesting that lower insulin levels (and high insulin receptor sensitivity) may be beneficial to extend lifespan [159,162,163].

## 12. How Can Hyperinsulinemia Be Modified?

In humans, there are at present three main strategies to prevent and manage hyperinsulinemia: reducing calorie intake, increasing hepatic insulin clearance and maximizing insulin sensitivity [164]. However, at this moment it is unclear which strategy is the best for preventing/managing hyperinsulinemia. Any dietary approach that causes weight loss improves hyperinsulinemia as body fat can only be stored, rather than oxidized in the presence of high insulin levels [164]. Only a few studies have studied the direct specific effects of a diet on hyperinsulinemia. Although a carbohydrate-restricted Mediterranean diet theoretically may confer the best effects, further research is needed to determine which diet is the best to modify hyperinsulinemia [164]. Studies during short-term very low calorie diets (VLCD) have found an increased hepatic insulin clearance and decline in plasma insulin concentrations, supporting that hepatic insulin clearance can be increased by energy restriction [165]. Furthermore, energy restriction induced by Roux-en-Y gastric bypass increased hepatic insulin clearance in obese subjects with normal glucose tolerance within 1 week [166]. Thus, insulin clearance can be modified within days by reducing energy intake. The early increases in insulin clearance after reduced energy intake result in metabolic changes typical for fasting (i.e., increased lipolysis and free fatty acid oxidation and a lower hepatic triglyceride content independent of weight loss) [109]. Interestingly, pharmacological lowering of hepatic triglyceride content in type 2 diabetes by rosiglitazone, a PPARγ receptor agonist, is also associated with a significant increase in insulin clearance within 16 weeks, and this effect is present without significant weight loss [167]. The pattern of food intake may also be important to reduce insulin levels. Five weeks of early time restricted feeding (6-hr feeding period during the day, with dinner before 3 pm) reduced insulin levels and improved β-cell responsiveness, insulin sensitivity, blood pressure and oxidative stress in prediabetic men even without weight loss [168]. Regular physical activity improves the whole-body metabolic health and can play a key role in the prevention and control of hyperinsulinemia, insulin resistance, prediabetes, type 2 diabetes and diabetes-related complications [169]. In a rodent study, exercise training prevented basal as well as glucose challenged insulin levels induced by a high-energy diet [170]. Two weeks of high-intensity interval or moderate-intensity continuous training improved β-cell function in people with prediabetes and type 2 diabetes [171,172]. Exercise training decreased pancreatic fat content and improved beta cell function regardless of baseline glucose tolerance in prediabetes and diabetes type 2 individuals [173]. By improving insulin sensitivity, increasing the production of glucose transporter-4 (GLUT-4) and lowering visceral adipose tissue, physical activity may further contribute to an improvement in hyperinsulinemia [172]. However, it remains an open question which form(s) of physical activity (resistance training, aerobic exercise or high-intensity interval training) is (are) the best to reduce hyperinsulinemia in humans, and further research is needed to find an answer on this question [164]. Pharmacological approaches that reduce insulin secretion and/or hepatic insulin clearance may be beneficial and prevent the progression to insulin resistance and hyperinsulinemia-associated conditions and diseases. Given the role of IDE in degrading insulin, the development of IDE activators for use in subjects with hyperinsulinemia may be a potential viable pharmacological approach [174]. The treatment of obese Zucker rats with diazoxide, an inhibitor of glucose-stimulated insulin secretion, decreased insulin secretion and increased insulin receptor binding, and this dual effect was associated with improved glucose tolerance and a decrease in weight gain in obese rats [175]. Furthermore, the administration of diazoxide to obese, nonketotic diabetes Otsuka Long-Evans Tokushima Fatty rats completely prevented the development of obesity and insulin resistance, and this was accompanied by a marked improvement in glucose tolerance and the disappearance of an exaggerated β-cell response to glucose in vitro [176]. Eight weeks of treatment of diazoxide to hyperinsulinemic obese humans induced greater attenuation of acute insulin responses to glucose and significant anti-obesity effects without inducing significant differences in insulin sensitivity and glucose levels [177]. Treatment with the somatostatin analogue octreotide-LAR q28d for 24 weeks suppressed insulin secretion in obese non-diabetic humans and this was associated with the loss of body weight and fat mass [178]. Metformin inhibits gluconeogenesis in the liver and delays glucose absorption from the gastrointestinal tract [179]. By reducing glucose load, metformin indirectly decreases endogenous insulin secretion. Interestingly, many epidemiological studies suggest a reduced incidence of cancer in patients treated with metformin compared to other antidiabetic therapies, and this may be related to metformin-mediated reductions in endogenous insulin secretion [180]. Sodium–glucose cotransporter 2 (SGLT2) inhibitors may also indirectly reduce hyperinsulinemia. Sodium–glucose cotransporter-2 inhibition by SGLT2 inhibitors leads to glycosuria and the lowering of plasma glucose [181]. One of the consequences of SGLT2 inhibitors is the development of a relative hypoinsulinemia, which is part of the first line of defense against hypoglycemia [182]. Interestingly, treatment with SGLT2 inhibitors also induces a marked reduction in cardiovascular risk and it has not been studied whether this may be related to the decrease in insulin levels [182]. Treatment with glucagon-like peptide-1 (GLP-1) receptor agonists may not only reduce plasma glucose levels and reduce energy intake, but also induce a normalization of the pulsatile pattern of insulin secretion and insulin sensitivity [183]. Both SGLT2 inhibitors and GLP-1 receptor agents reduce (due to its reducing effects on hyperinsulinemia?) body weight, visceral fat mass, blood pressure, and improve lipid profile and cardiovascular outcomes [184]. On the other hand, some other frequently used antidiabetic drugs do not result in the restoration of a normal pulsatile insulin response to a glucose load (e.g., sulphonylureas and long-acting insulin preparations) and their use may even contribute to the development of tissue insulin resistance and glucose intolerance [185,186]. At the moment, there is a lack of evidence showing that reducing hyperinsulinemia before or early in the development of insulin resistance has indeed long-term beneficial effects on health in humans. Future research should focus on developing (new) strategies/drugs that can successfully prevent, delay or mitigate hyperinsulinemia and thereby hyperinsulinemia-mediated pathologies.

## 13. Concluding Remarks

Hyperinsulinemia precedes insulin resistance and may already be present in subjects with normal glucose tolerance. Genes, consumption of the “modern” Western diet, over-nutrition and other environmental factors may increase insulin secretion, decrease insulin pulses and/or reduce hepatic insulin clearance and thereby cause hyperinsulinemia. Hyperinsulinemia disturbs the balance of the insulin–GH–IGF axis and this causes a shift in the insulin : GH ratio towards insulin and away from GH. This shift in the insulin : GH ratio blocks lipid breakdown and promotes further energy storage and lipid synthesis, resulting in obesity due to more fat accumulation and a lower energy expenditure. There is considerable evidence that hyperinsulinemia is the common etiological factor in the development of metabolic syndrome, type 2 diabetes, cardiovascular disease, cancer and premature mortality and also plays an essential role in age-related decline. Therefore, interventions that reduce hyperinsulinemia might play a key role in the prevention and treatment of age-related decline, obesity, type 2 diabetes, cardiovascular disease and cancer. An important component of future research should be to study which (new) strategies are the best for preventing/managing hyperinsulinemia and to investigate whether preventing/reducing hyperinsulinemia before or early in the development of insulin resistance has indeed beneficial effects on health and longevity.

## Figures and Tables

**Table 1:** The three phases of the “Food and Famine” theory.

Time Course	Major Fuel Used	Hormonal Control
Immediate Postprandial Period	Stimulation of glucose uptake Stimulation of glycogenesis Stimulation of amino acid uptake Stimulation of free fatty acid uptake Stimulation of lipogenesis	Insulin
Early Postprandial Period	Stimulation of amino acid uptake Stimulation of protein synthesis Reduction in insulin resistance Suppression of proteolysis	Insulin, GH + IGF-I
Late Postprandial Period	Reduction in insulin resistance Inhibiting of insulin release Stimulating of lipolysis Stimulating lipid oxidation Reduction in protein oxidation	IGF-I IGF-I IGF-I (indirectly) by reducing insulin secretion GH + IGF-I

**Table 2:** Hyperinsulinemia at baseline is positively associated with the development of many conditions and diseases at follow-up.

	Reference No	Type of Study
Obesity	4	Prospective, observational
	5	Prospective, observational
	116	Prospective, observational
Impaired Glucose Tolerance	119	Prospective, observational
	126	Prospective, observational
	127	Prospective, observational
Type 2 Diabetes	9	Prospective, observational
	84	Prospective, observational
	86	Prospective, observational
	120	Prospective, observational
	124	Prospective, observational
	127	Prospective, observational
Cardiovascular Disease	1	Prospective, observational
	2	Prospective, observational
	3	Prospective, observational
	130	Prospective, observational
	131	Prospective, observational
	132	Prospective, observational
Cancer		
Liver Cancer	6	Prospective, observational
Colorectal Cancer	8	Prospective, observational
Colorectal Cancer	145	Prospective, observational
Pancreas	145	Prospective, observational
Breast Cancer	146	Prospective, observational
Cancer Mortality	7	Prospective, observational
Decreased Survival	164	Prospective, observational

## Data Availability

Not applicable.
